# Supplementary material for: Ezrin enhances line tension along transcellular tunnel edges via NMIIa driven actomyosin cable formation
Source: Nat Commun. 2017 Jun 23;8:15839. doi: 10.1038/ncomms15839 (PMC5490010; doi:10.1038/ncomms15839)
Supplement: Supplementary Information — Supplementary Figures and Supplementary Note [file ncomms15839-s1.pdf]

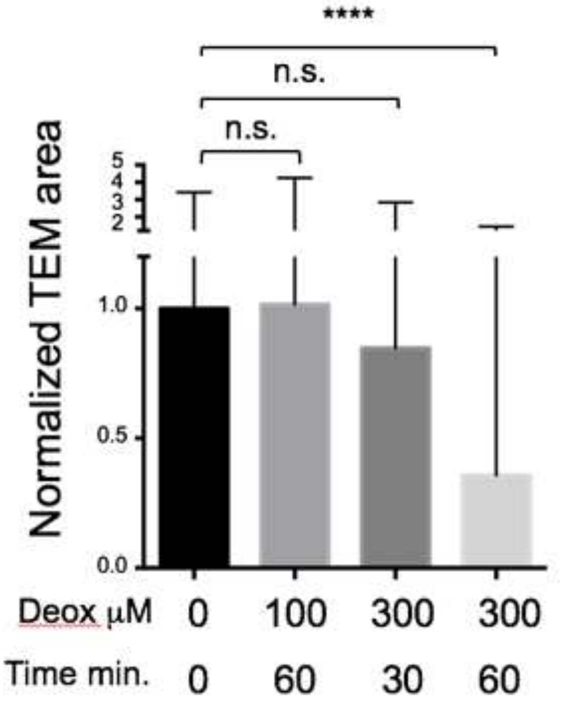

Supplementary Figure 1: Deoxycholic acid treatment decreases TEM size.

HUVECs seeded on coverslips were treated with 50µg/ml exoC3 overnight, followed by treatment with 100µM or 300µM deoxycholic acid (Deox) for 30 and 60 minutes, as indicated. Cells were then stained with FITC-conjugated phalloidin. Images were taken with Leica droit DM5500 and area of the TEMs measured with Fiji ImageJ. Histogram shows mean values ± SEM, *n*=30 each condition, 2 replicas. Unpaired, two-sided Student's t-test (****p < 0.0001 or ns > 0.05).

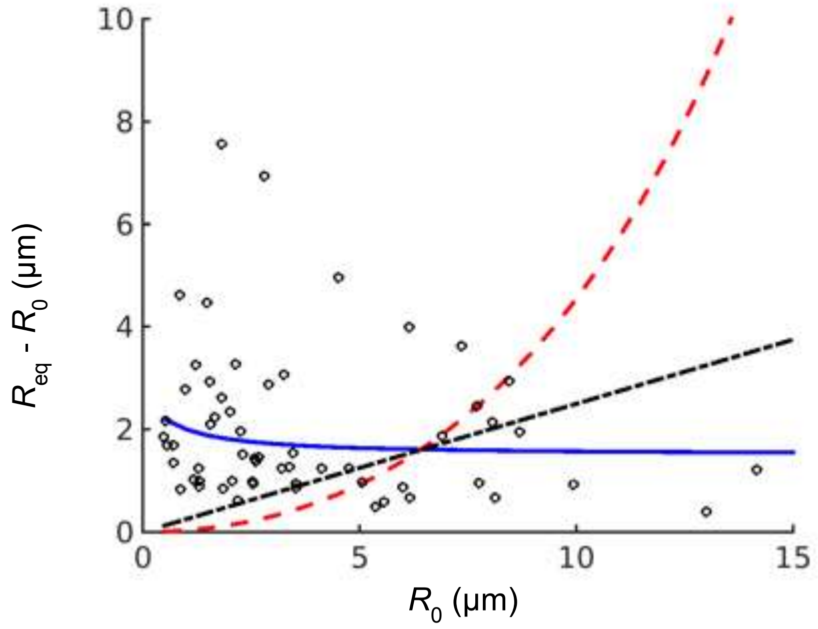

Supplementary Figure 2: Alternative physical models

Comparison between the predictions of the alternative physical models discussed in the text and the experimental data of TEM radius increase after ablation, $R_{eq} - R_0$, versus initial radius, R_0. Black circles correspond to experimental data, the thick blue line is the proposed model with a time-increasing line tension, the dashed red line is an alternative model where TEM opening is limited by the bending resistance of the cable, and the dash-dotted black line is another alternative model where line tension is restored by convection of molecular components towards the cable.

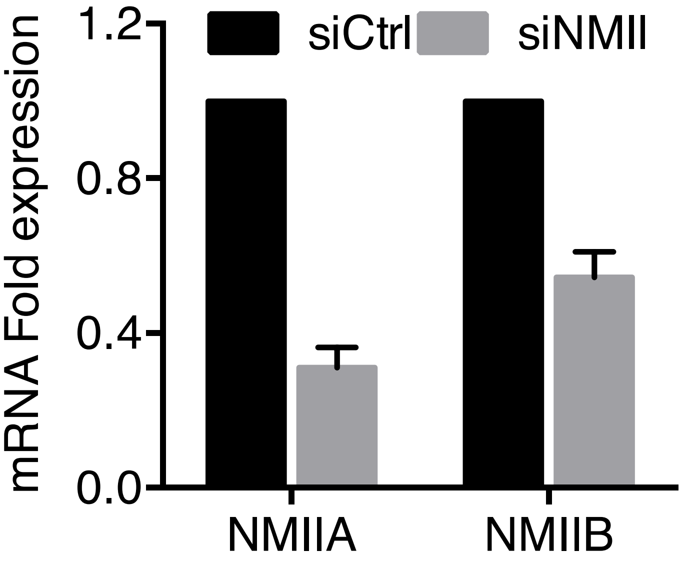

Supplementary Figure 3: Control of siRNA NMII

HUVECs treated 48 h with NMII siRNA. NMII knockdown was monitored by qRT-PCR, normalized to RPLC0 RNA and expressed relative to siRNA control (*n*=3; mean ± SEM). RNA were purified using QIAGEN RNeasy Kit and 1µg of RNA were used to produce cDNA with High capacity RT kit (Life technologies). The qPCR reaction was performed using SYBR Green (Life technologies) on AB7500. Primers for NMIIA/MYH9: CCTCAAGGAGCGTTACTACTCA and CTGTAGGCGGTGTCTGTGAT, primers for NMIIB/MYH10: AAAAGTCTCGTGCTGTTCGTC and TGTCCCGGAATAGGAATA TAGCC, primers for RPLC0: TGCATCAGTACCCCATTCTATCAT and AAGGTG TAATCCGTCTCCACAGA.

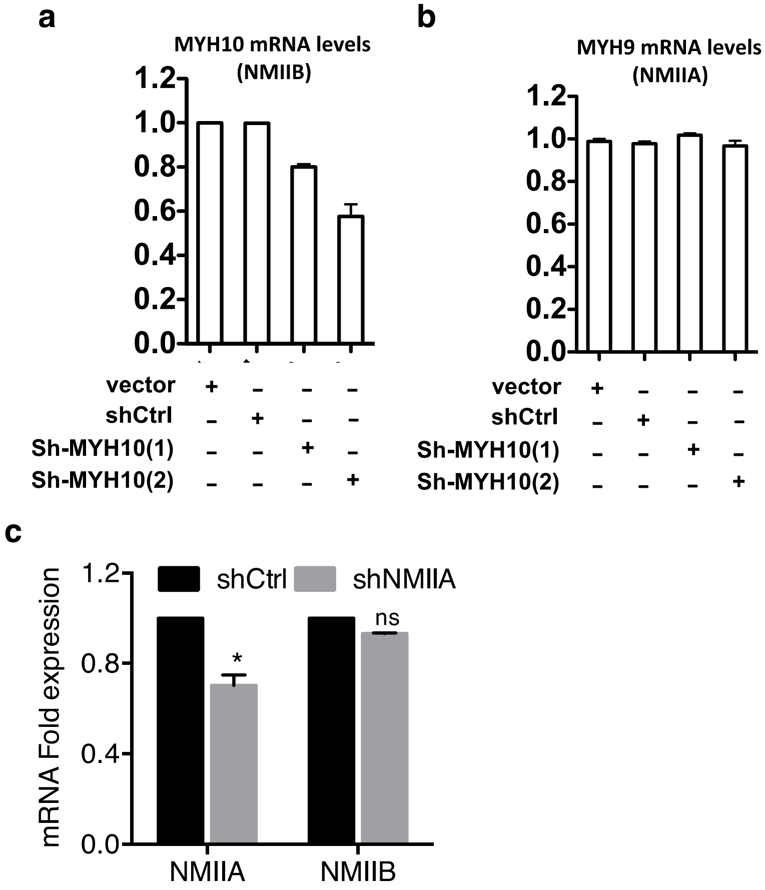

Supplementary Figure 4: control of shRNA NMIIa and NMIIb

a-c) HUVECs treated 48 h with shNMIIA or shNMIIB. NMII knockdown was monitored by qRT-PCR, normalized to RPLC0 RNA and expressed relative to shRNA control (n = 3; mean ± SEM). RNA were purified using QIAGEN RNeasy Kit and 1µg of RNA were used to produce cDNA with High capacity RT kit (Life technologies). The Q-PCR reaction was performed using SYBR Green (Life technologies) on AB7500. Primers for NMIIA/MYH9: CCTCAAGGAGCGTTACTACTCA and CTGTAGGCGGTGTCTGTGAT, primers for NMIIB/MYH10: AAAAGTCTCGTGCTGTTCGTC and TGTCCCGGAATAGGAATATAGCC, primers for RPLC0: TGCATCAGTACCCCATTCTATCAT and AAGGTGTAATCCGTCTCCACAGA.

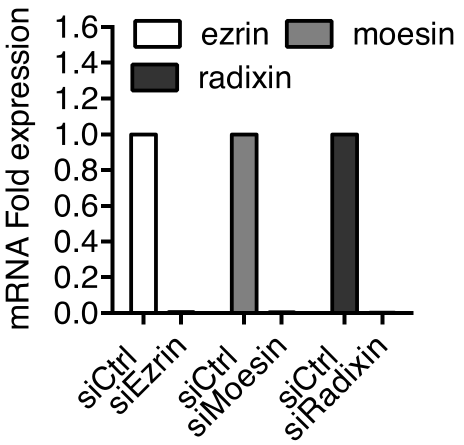

Supplementary Figure 5: control of siRNA ezrin, radixin, moesin

HUVECs treated 48 h with ezrin, radixin or moesin siRNA (Dharmacon). ERM knockdown was monitored by qRT-PCR, normalized to RPLC0 RNA and expressed relative to siRNA control (*n*=3; mean ± SEM). RNA were purified using QIAGEN RNeasy Kit and 1µg of RNA were used to produce cDNA with High capacity RT kit (Life technologies). The Q-PCR reaction was performed using SYBR Green (Life technologies) on AB7500. Primers used were for ezrin: ATGCCCCACGTCTGAGAATC and TCCTGCGGCGCATATACAAC, primers for radixin: AATCGACAAAAAGGCACCT GA and CCATACATAAGGCCAAAATCCGC, primers for moesin: GCTGTCCAGTCTAA GTATGGCG and TGCGGGAGCAACTTGTCTC. primers for RPLC0: TGCATCAGTACCCCATTCTATCAT and AAGGTGTAATCCGTCTCCACAGA.

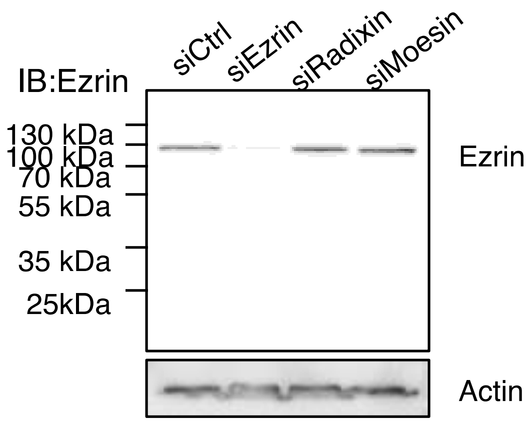

Supplementary Figure 6: control of siRNA ezrin, radixin, moesin

HUVECs treated 48 h with ezrin, radixin or moesin siRNA ON-TARGETplus SMARTpool (Dharmacon). Ezrin knockdown was monitored by immunoblotting showing the specificity of siEzrin on ezrin protein level compared to siRadixin and siMoesin. Immunoblot anti-actin show equal loading (sigma).

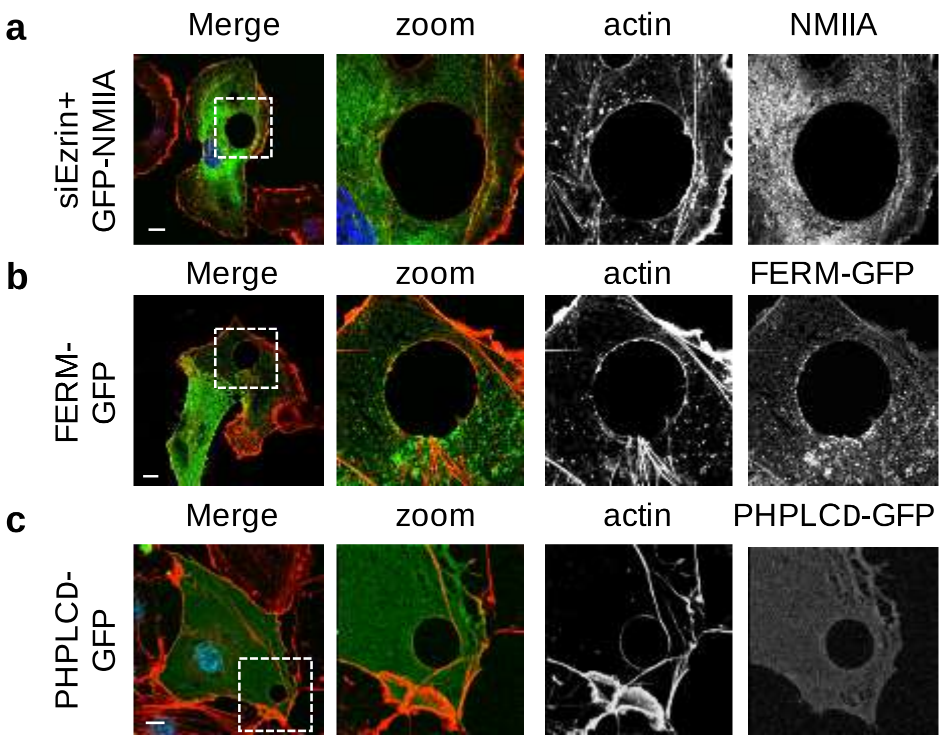

Supplementary Figure 7: Localization of markers along TEM edges

a) HUVECs were transfected with siEzrin and NMIIA-GFP expressing vector. Transfected cells were next treated 24 h with exoC3. Actin was labeled with phalloidin (red). Scale bar, 10 µm. **b)** HUVECs were transfected with ezrin-FERM-GFP expressing plasmid. Transfected cells were next treated 24 h with exoC3. Actin was labeled with phalloidin (red). Scale bar, 10 µm. **c)** HUVECs were transfected with expression plasmid encoding a pleckstrin homology domain (PH) of PLCδ fused to GFP. Transfected cells were next treated 24 h with exoC3. Actin was labeled with TRITC-conjugated phalloidin (green). Scale bar, 10 µm.

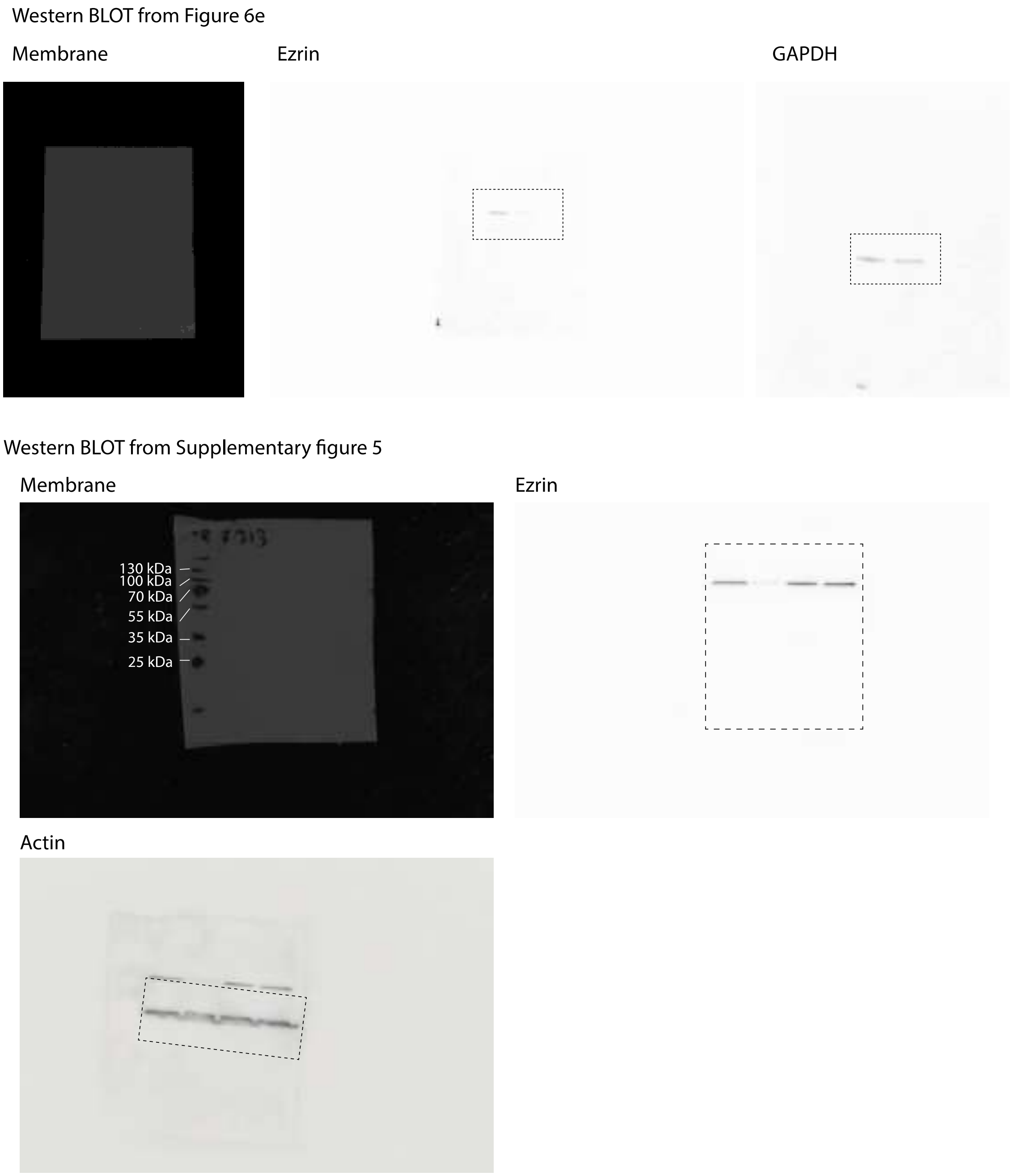

Supplementary Figure 8: Full size images of all western Blot

Supplementary Note 1: Alternative mechanisms governing TEM stabilization

Using analytical modeling, we investigate possible physical mechanisms underlying TEM stabilization upon ablation of the actin cable. One first possibility is that TEM opening after ablation is resisted by the bending rigidity of the remaining actin cable. In this scenario, a new equilibrium would be reached when bending resistance, which scales with $1/R_0 - 1/R$, balances membrane tension. However, one can readily show that the resulting radius increase $\Delta R = R_{eq} - R_0$ would depend on the initial radius R_0 (Supplementary Fig. 2). Such prediction is in conflict with our experimental observations that $\Delta R = 2\ \mu\text{m}$, independently of R_0 (Fig. 2b). Thus, we conclude that bending resistance of the remaining cable cannot be the dominant mechanism that stabilizes TEMs after ablation. The second possibility is that TEMs are ultimately stabilized by the reorganization of an actin cable around the rim, which eventually becomes strong enough to balance membrane tension. Physically, this can be interpreted as a rebuild of line tension after laser ablation as the cable reforms. To shed light on the molecular mechanisms underlying the reassembly of the actin cable, we consider different possible hypotheses that we tested with regards to our experimental data on TEM radius after ablation. First, we can hypothesize that line tension builds up due to molecular components being recruited from the cytoplasm to the cable by the sweeping motion of the TEM rim, thus with convection being the limiting mechanism. In this scenario, molecules are recruited over the area swept by the rim and distributed over the rim's length. Thus, line tension increase would scale as $T \sim (R^2 - R_0^2)/(R - R_0)$. However, this convective hypothesis leads to a prediction for ΔR that increases with the initial radius R_0, which is again inconsistent with our experimental data (Supplementary Fig. 2). This does not exclude that a convective mechanism contributes to F-actin recruitment around TEMs; however, it precludes F-actin convection to be the critical limiting factor pointing for the likely importance of a F-actin crosslinking mechanism. Second, we can suppose that line tension develops over the typical time required for the organization of the actin cable and find which time dependence T=$T(t)$ would be compatible with our measurements. The precise form of this dependence will be indicative of the dominant mechanism behind actin cable assembly: a dependence of the form $T \sim \sqrt{t}$ would

correspond to diffusion of the cable components towards the rim, and a linear dependence $T \sim t$ to a constant-rate cable strengthening by actin polymerization, reorganization, cross-linking, or a combination thereof. In the main text, it is mathematically shown that a linear dependence $T \sim t$ is appropriate to describe the experimental results suggesting that line tension build up occurs by constant-rate cable strengthening by actin polymerization.
